# Supplementary material for: The Different Composition of Coumarins and Antibacterial Activity of Phlojodicarpus sibiricus and Phlojodicarpus villosus Root Extracts
Source: Plants (Basel). 2024 Feb 22;13(5):601. doi: 10.3390/plants13050601 (PMC10933757; doi:10.3390/plants13050601)
Supplement: Supplementary file 1 [file plants-13-00601-s001.zip › plants-2818198-supplementary.pdf]

Table S1. The functional group structures.

$C_2H_3O_2$	$C_4H_7O_2$	$C_5H_7O_2$	$C_5H_9O_2$
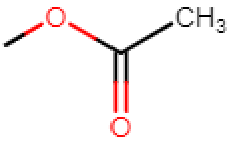	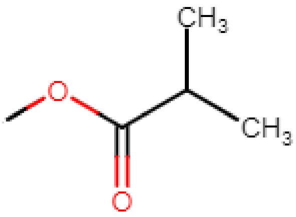	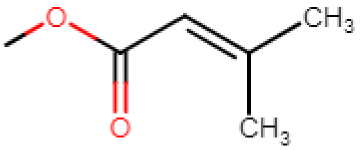	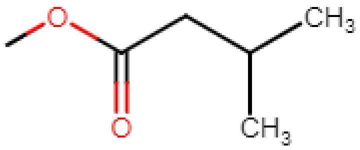
acetyloxy	isobutyryloxy (isobutyroyloxy)	senecioyloxy	isovaleryloxy (isovaleroyloxy)
	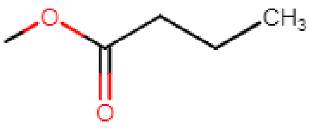	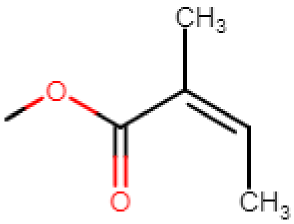	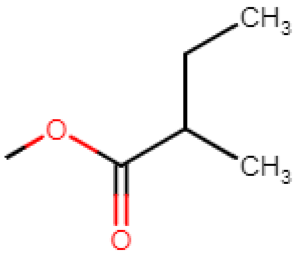
	butoxy	angeloyloxy	2-methylbutyroyloxy
		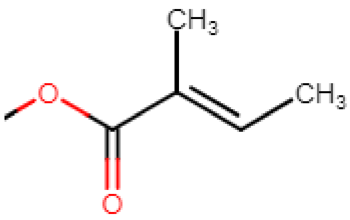	
		tigloyloxy	
